# Electronic Health Record-Related Safety Concerns: A Cross-Sectional Survey of Electronic Health Record Users

**DOI:** 10.2196/medinform.5238

**Published:** 2016-05-06

**Authors:** Sari Palojoki, Tuuli Pajunen, Kaija Saranto, Lasse Lehtonen

**Affiliations:** ^1^ University of Eastern Finland Faculty of Social Sciences and Business Studies, Department of Health and Social Management Kuopio Finland; ^2^ Helsinki and Uusimaa Hospital District, Helsinki University Hospital Group Administration Helsinki Finland; ^3^ FCG Consulting Ltd. Helsinki Finland

**Keywords:** Electronic Health Records, Health Information Technology, Patient Safety, Risk Assessment, Questionnaire

## Abstract

**Background:**

The rapid expansion in the use of electronic health records (EHR) has increased the number of medical errors originating in health information systems (HIS). The sociotechnical approach helps in understanding risks in the development, implementation, and use of EHR and health information technology (HIT) while accounting for complex interactions of technology within the health care system.

**Objective:**

This study addresses two important questions: (1) “which of the common EHR error types are associated with perceived high- and extreme-risk severity ratings among EHR users?”, and (2) “which variables are associated with high- and extreme-risk severity ratings?”

**Methods:**

This study was a quantitative, non-experimental, descriptive study of EHR users. We conducted a cross-sectional web-based questionnaire study at the largest hospital district in Finland. Statistical tests included the reliability of the summative scales tested with Cronbach’s alpha. Logistic regression served to assess the association of the independent variables to each of the eight risk factors examined.

**Results:**

A total of 2864 eligible respondents provided the final data. Almost half of the respondents reported a high level of risk related to the error type “extended EHR unavailability”. The lowest overall risk level was associated with “selecting incorrectly from a list of items”. In multivariate analyses, profession and clinical unit proved to be the strongest predictors for high perceived risk. Physicians perceived risk levels to be the highest (*P*<.001 in six of eight error types), while emergency departments, operating rooms, and procedure units were associated with higher perceived risk levels (*P*<.001 in four of eight error types). Previous participation in eLearning courses on EHR-use was associated with lower risk for some of the risk factors.

**Conclusions:**

Based on a large number of Finnish EHR users in hospitals, this study indicates that HIT safety hazards should be taken very seriously, particularly in operating rooms, procedure units, emergency departments, and intensive care units/critical care units. Health care organizations should use proactive and systematic assessments of EHR risks before harmful events occur. An EHR training program should be compulsory for all EHR users in order to address EHR safety concerns resulting from the failure to use HIT appropriately.

## Introduction

Previous success in the adoption and use of health information technology (HIT) has been darkened by the growing number of reports of its unintended consequences and potential for errors [1]. Risks associated with electronic health records (EHR) have been identified as related to technologies themselves, their applications, and their use [2]. The systematic analysis of EHR-related safety concerns is clearly a prerequisite for recognizing safety threats [3,4]. The sociotechnical approach facilitates understanding of the risks in the development, implementation, and use of EHR and HIT while accounting for complex interactions of technology within the health care system [5-12].

Sittig and Singh have provided extensive work and a foundation for understanding EHR safety. These researchers define the HIT work system as the combination of the hardware and software required to implement HIT, as well as the social environment in which it is implemented [6-8]. According to Sittig and Singh’s research, HIT errors may involve failures of either structures or processes. These errors can occur in the design and development, implementation and use, or evaluation and optimization phases of the HIT lifecycle [9]. HIT-related errors occur anytime the HIT system is unavailable for use, malfunctions during use, is used incorrectly, or interacts with another system component which incorrectly results in data loss or incorrect entry, display, or transmission. The dimensions are not independent, sequential, or hierarchical, but rather interdependent and interrelated concepts similar to the compositions of other complex adaptive systems [6-8]. This approach is consistent with the currently recommended approaches to systems and human factors used to identify and minimize error [9]. HIT errors should be defined from the socio-technical viewpoint of end users [6-8].

Risk assessment is the process through which organizations develop an understanding of the risks they face [13]. This process is supported by various tools and techniques. Risk analysis consists of determining the consequences and their probabilities for identified risk events. The consequences and their probabilities are then combined to determine a level of risk [14]. Use of a risk assessment matrix is a growing practice. The simplicity and ease of use of this approach contributes to widespread adoption, including a generic international standard for risk assessment techniques to support risk management [13]. Organizations can reduce the number and severity of EHR-related safety events by anticipating the risk factors [15].

The results of a recent study suggest that EHR safety depends on persistent testing and monitoring, especially in terms of the ongoing appraisal of sociotechnical factors that affect the use and maintenance of EHRs. Because the new EHR adopters lack relevant skills and resources, it is more critical to develop techniques to support awareness of the risks, as well as their monitoring and management [16]. One method to support awareness of risks is to identify risk indicators that are easily detectable. Sittig and Singh present a red-flag-based approach that can serve to identify potential EHR safety concerns. Common EHR-related safety concerns have been identified based on Sittig and Singh’s work in EHR-related patient safety, and a survey focusing on the frequency of serious EHR-related safety events, variables affecting serious EHR-related safety events, and the tracking of EHR-related safety measurements [15,16].

The research data in this study has been refined to explore users’ perceptions of high- and extreme-risk severity ratings in the use of EHR. We were interested in assessing EHR users’ perceptions of EHR safety issues because no previous study has explored this problem area in a specialized hospital context. Consequently, we used a mixed-methods approach in several phases to develop and validate a questionnaire based on Sittig and Singh’s research and findings [15,16]. The final Finnish questionnaire consisted of eight error types, each with three to six related questions. Future research will focus on developing a tool to mitigate EHR-related safety concerns.

## Methods

### Research Questions

Our goal was to study health care professionals’ perceptions of common EHR concerns. The specific objective was to concentrate on severe-risk error types and risk factors.

This study aimed to answer the following questions:

1. Which of the common EHR error types are associated with perceived high- and extreme-risk severity ratings among EHR users?
2. Which variables are associated with high- and extreme-risk severity ratings?

### Recruitment

This study was a quantitative, non-experimental descriptive study of Finnish EHR users. A cross-sectional web-based questionnaire study took place over a four-week time period in the beginning of 2015. The study was conducted in the Hospital District of Helsinki and Uusimaa, and included 23 hospitals (covering a population of 1.6 million Finns; 34% of the Finnish population) that treat half a million patients annually. The hospital district runs the largest academic teaching hospital (Helsinki University Hospital) in Finland, which covers all medical specialties and emergency services in its different facilities. Furthermore, the district runs four regional hospitals that support local primary care outside the Helsinki metropolitan area. The entire hospital district has approximately 22,300 employees [17].

All nurses, nursing aids, physicians, clinical secretaries, and academic hospital workers (eg psychologists, pharmacists and clinical nutritionists) working, and potentially using the EHR, throughout the hospital district comprised the target population. The qualifications of health care professionals in Finland, as in other member states of the European Union (EU), are in accordance with the EU directive on professional qualifications (2005/36/EC) [18]. This directive applies to doctors, specialist doctors, nurses, specialist nurses, and midwives. There are no set entry requirements for clinical secretaries, but they do require proficient information technology (IT) skills to use and process EHRs.

These hospitals have used the same EHRs for several years. The hospital district has a computerized physician order entry with clinical decision support and major ancillary systems (ie laboratory), a picture archiving and communication system, as well as a clinical data repository for reviewing results. The closed loop medication system is not part of the EHRs. These hospitals have the same risk-assessment approach and systematic education for all clinicians as part of their patient safety programs.

The questionnaire took place in early 2015. At the same time, a new version of the EHR program was implemented in order to incorporate the system into the Finnish national health care archive, known as KanTa. Although the overall availability of EHR in 2014 was as high as 99.9%, the system’s total unplanned widespread unavailability for12.4 hours during 2014 threatened the continuity of operations in these hospitals.

A commercial online platform (Webpropol) served to conduct the survey. We sent the questionnaire, with detailed information for answering, as well as an explanation of the risk matrix, to all potential EHR users (N=17,336) at the same time. Identifying exactly which individuals use EHR was impossible, so questionnaires were sent to all professionals in these groups. We also advised the participants to rate all error types and risk factors on the questionnaire in their own working environment during the last 12 months. We sent two reminder e-mails to all individuals who had not completed the questionnaire.

The organization’s research review process approved the study protocol. Since patients were not the subject of this study, Finnish national legislation (488/199) did not require the approval of the Institutional Review Process for the study [19]. All respondents will remain anonymous, and the study involved no financial incentives.

### Questionnaire Items and Assessment Scale

The questionnaire consisted of eight error types based on Sittig and Singh’s previous research [7,15,16]. Each of the error types included three to six EHR-related safety issues or risk factors based on commonly identified EHR safety concerns.

The error type *incorrect patient identification* includes questions related to key patient-identifying information. These errors include information missing from the EHR screens or printouts, the absence of documented processes and procedures for verifying patient identification at crucial stages of patient visits, and incorrect site information or incorrect patient surgery/procedure information originated from an order that was entered for the wrong patient. One commonly recognized safety issue, in which nurses use copies of one or more patient barcode identification bands taped to their clipboard as a workaround when performing barcoded medication administration, was omitted during questionnaire development because this practice does not exist in these hospitals’ EHRs.

The error type *extended EHR unavailability* means that some portion or, more likely, all of the patient’s medical records are unavailable for review. This error results from total or partial failure of the EHR system, or planned downtime.

*Failure to heed a computer-generated warning or alert* is an error type in which critical information, even if sent to the correct person at the right time and displayed prominently on the computer screen, can be overlooked due to an overabundance of other false-positive information. This error means that items can potentially indicate the existence of a given condition when this is actually not the case. Overlooked data such as these can lead to erroneous or delayed diagnoses or treatments.

*System-to-system interface errors* are the result of communication problems between applications. These errors can prevent data from one application (eg a laboratory system) from reaching another application (eg the EHR), or corrupt the data itself.

*Failure to find or use the most recent patient data* errors can cause clinicians to make erroneous clinical decisions and lead to incorrect, unnecessary or delayed tests, procedures, or therapies. Such failures usually result from difficulties navigating, viewing, understanding, or interacting with user interfaces.

The error type *EHR time measurement translational challenge* occurs when the computer cannot properly translate time measurements as EHR users understand and enter them. Examples of consequences associated with this error type include routine tests, medications, or procedures that can be ordered *daily*, yet continue long after they are clinically needed because the order lacked a stop date.

*Incorrect item selected from a list of items* is an error type that occurs when an EHR user inadvertently selects a listed item that appears directly over the item the user intended to select. Such errors can occur if the user fails to notice or understand the difference between items, or simply selects the incorrect item. *Open, incomplete or missing orders* can result from failure to complete the order entry process, including signing and submitting the orders.

Health care failure mode and effects analysis (HFMEA) is a technique for preventing process and product problems before they occur. HFMEA focuses on what problems could occur, as well as their severity [20]. The HFMEA approach entails the prioritization of potential risks by determining the severity and probability of a failure mode [21]. The questionnaire scale in this study was based on the qualitative risk matrix after consulting with a professor of risk assessment research. The basic structure of the risk matrix is consistent with a widely adopted concept of risk and consists of one axis representing categories of probability (likelihood or frequency) of possible hazardous events, while the other axis represents categories of severity (impact or consequences) of those events (ie how often do these things happen, and how bad are they when they occur?). Each intersecting cell of the matrix (ie column-row pair) is pre-assigned an overall risk severity as insignificant, low-, medium-, high-, and extreme-risk. The questionnaire scale consisted of these values, with *insignificant* corresponding to a value of 1 and *extreme-risk* to 5 [22,23].

### Statistical Analyses

We sent the questionnaire to every potential EHR user, encompassing all staff members in the hospital district’s 23 hospitals. Previous data on personnel absenteeism of the 17,336 total staff members indicated that at least 10% of them would be on different kinds of leave (eg sick, study, maternity, parental, or research leave) and thus ineligible to participate in the survey.

Of the 15,602 eligible respondents, 2868 completed the survey, yielding an overall response rate of 18.38%. Of the 2868 respondents, 4 were eliminated due to missing data on all but a few questions, leaving a final dataset of 2864 respondents. To assess the representativeness of the sample, we gathered the sex, age, profession and education distributions of all staff members from the hospital district’s centralized human resources (HR) systems’ personnel records, and used χ2 tests to compare the corresponding sample distributions between participating and non-participating employees. Despite the relatively low response rate, comparison of the respondents’ background characteristics to personnel department data on all staff members revealed only a few significant demographic differences between participating and non-participating employees. We also collected information on the respondents’ sex, age, profession and education distributions from the HR systems and used χ2 tests to compare the corresponding sample distributions. The sex, age and education distributions did not differ in a statistically significant manner from the staff records. Registered nursing professionals and medical doctors, compared to other professionals, were slightly overrepresented in the sample (*P*<.001). However, this was not considered problematic, since only respondents who did not use the EHR were asked not to answer the questionnaire, and non-users consisted mainly of professionals other than nurses and doctors (eg administrative department staff).

The dependent variables were based on the eight multi-item scales described above, each having between three and six individual question items. We tested the reliability of the summative scales with Cronbach’s alpha. All of the dimensions showed good internal reliability, with alpha values ranging from .789 to .888 (see Multimedia Appendix 1). For the statistical analyses, we regrouped each of the multi-item scales into binary variables. After the preliminary analyses, we decided to define the outcome variable as responses of “Poses a high risk” (value 4 on a scale from 1 to 5) or “Poses an extreme risk” (value 5 on a scale from 1 to 5) to any of the items on the subscale. We chose this cut-off point because reporting a severe risk related to patient care was considered an important indicator of patient safety. Logistic regression served to assess the association of the independent variables to each of the eight risk factors.

After initial univariate models and model selection using backward variable selection, including all of the available independent variables, only the following information about a respondent figured in the final multivariate models: profession, type of clinical unit, professional experience (in years), EHR training mode (type of EHR training received , such as classroom training or eLearning) and self-reported EHR skills (assessed on a scale of 4 to 10 and regrouped into three groups labeled *poor*, *fair*, and *good*). In the models, we included variables at *P*<.10 level of significance, and a 95 % confidence level was used to calculate CIs. We used statistical software R version 3.1.2 to carry out all statistical analyses [24].

## Results

### Respondents’ Characteristics and Perceived Risk Level

The final dataset consisted of 2864 eligible respondents, 85.16% (2439/2864) of whom were women and 77.72% (2226/2864) of whom were aged 34 years or older. The participants were primarily nursing professionals (71.37%, 2044/2864) and held a university of applied sciences or equivalent degree (56.81%, 1627/2864); 15.12% (433/2864) were physicians. As expected, the largest proportion of participants (57.19%, 1638/2864) worked in a ward or outpatient clinic.

Of the respondents, 92.18% (2640/2864) used EHRs several times per shift. An additional 3.00% (86/2864) of the respondents said they consulted the EHR system once or twice per shift, while 1.01% (29/2864) of the respondents did not use the EHR themselves, but acted as the superior of other EHR users and consequently were aware of EHR risk factors.

A total of 30.73% (880/2864) of the respondents had participated in EHR eTraining, 28.04% (803/2864) attended a general lecture about EHR, and 21.30% (610/2864) received classroom training; 10.61% (304/2864) received personal guidance or training from an IT support person.

The distribution of background variables and the percentage of respondents reporting a high- or extreme-risk rating per error type (defined as reporting a high or extreme risk level on at least one subscale item) appears in Multimedia Appendix 2.

The highest proportion, nearly half of the respondents in both gender groups (48.99%, 1403/2864), reported a high-risk level related to *extended EHR unavailability*. A high perceived risk was reportedly related to *incorrect patient identification*, *system-to-system interface errors*, *failure to find or use the most recent data*, *EHR time measurement errors*, and *open/incomplete orders*. The lowest overall risk level was associated with *selecting an incorrect item from a list of items* (27.02% [659/2439] of females and 32.94% [140/425] of males). Men reported higher levels of perceived risk scores than did women. Older respondents tended to report higher risk levels, but the association was inconsistent across all error types.

Physicians reported higher risk levels on all of the eight factors, especially those relating to *extended EHR unavailability* and *failures to find the most recent patient data*. Registered nursing professionals reported the second highest overall risk scoring, and the highest values were related to *extended EHR unavailability* and *open/incomplete or missing orders*. Clinical clerks and academic specialists reported lower risk levels than did other professionals. Clinical clerks’ highest perceived scoring was related to *extended EHR unavailability*, whereas academic specialists’ highest values were related to *failure to find or use the most recent patient data* and *system-to-system interface errors*.

Emergency departments (ED), operating rooms (OR), and procedure units were associated with higher perceived risk levels, whereas clinical laboratory and radiology units were related to lower risk scoring. Professionals working on general wards reported high-risk scoring on *extended EHR unavailability*, *failure to find or use the most recent patient data*, and *open, incomplete or missing patient data*.

Having received no EHR training was associated with higher perceived risk levels, and classroom and eLearning correlated with lower risk levels. However, we found no differences in the error type relating to *system-to-system interface errors*. Poor self-reported EHR skills were related to high perceived risk.

### Factors Associated with Perceived Risk Ratings in Multivariate Logistic Regression Analyses

The initial univariate analyses (results not shown) found profession and clinical unit to be the strongest predictors for perceived high- and extreme-risk ratings. Physicians reported a higher perceived risk on all risk dimensions (odds ratios between 1.21 and 2.55). The associations remained statistically significant in the multivariate analyses, even after adjusting for education, work experience, type of EHR training received, and self-reported EHR skills for all of the risk factors, except the one related to *incorrect patient identification* (odds ratios between 1.30 and 2.51). Academic specialists reported lower levels of perceived risk, and the association remained significant in multivariate models of four of the eight risk levels measured.

Health care professionals working in EDs, ORs, and procedure units reported higher perceived risk ratings on all error types. The association remained robust for most dependent variables, even after adjusting for profession and other background variables. Professionals working at an intensive care unit (ICU)/critical care unit (CCU) reported higher perceived risk ratings on *extended EHR unavailability*, *system-to-system interface errors* and *open, incomplete or missing orders*, but in the multivariate models the association remained significant only for *interface* errors. Lower perceived risk levels were associated with working in a clinical laboratory or in radiology, providing less acute patient care, and working in outpatient units, although to a somewhat lesser degree.

Prior participation in eLearning courses on EHR-use was associated with lower risk ratings on some of the risk factors (*extended EHR unavailability*, *P*=.03; *EHR warning dismissed*, *P*=.015; *failure to find or use the most recent patient data*, *P*=.018). General lecture training was associated with greater risk, although the association did not remain significant in most of the multivariate models. As expected, poor self-reported EHR-use skills were associated with higher risk ratings, and the effect remained significant even after controlling for other factors. However, controlling for the level of EHR-use skills in multivariate models failed to explain the association of the other factors with the risk dimensions. The association of background variables with perceived EHR risk rating appears in Multimedia Appendix 3.

**Figure 1:**
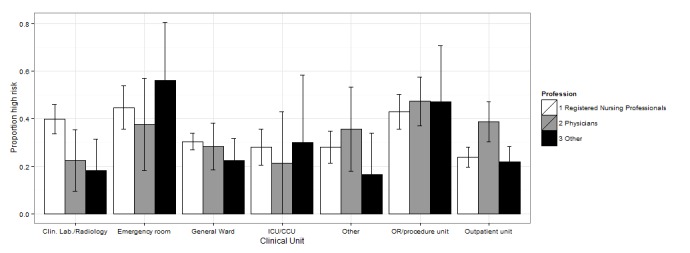
Proportion of high risk according to respondents’ professions and clinical unit (+95% CIs) in incorrect patient identification.

We also tested the interaction between professional qualification and working unit. The interaction terms did not remain significant in the multivariate analyses, in large part due to small sample sizes in some of the subgroups. To analyze the joint association between profession and clinical unit, we combined academic specialists and clinical clerks into one group and assigned labor wards to the *other units* group (see Figure 1 and Multimedia Appendix 4 for margins of error and 95% CIs).

**Figure 2:**
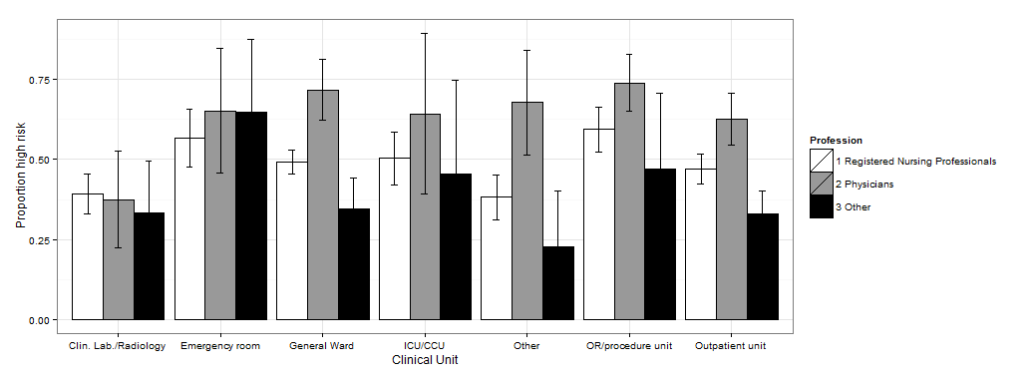
Proportion of high risk according to respondents’ professions and clinical unit (+95% CIs) in extended EHR unavailability.

**Figure 3:**
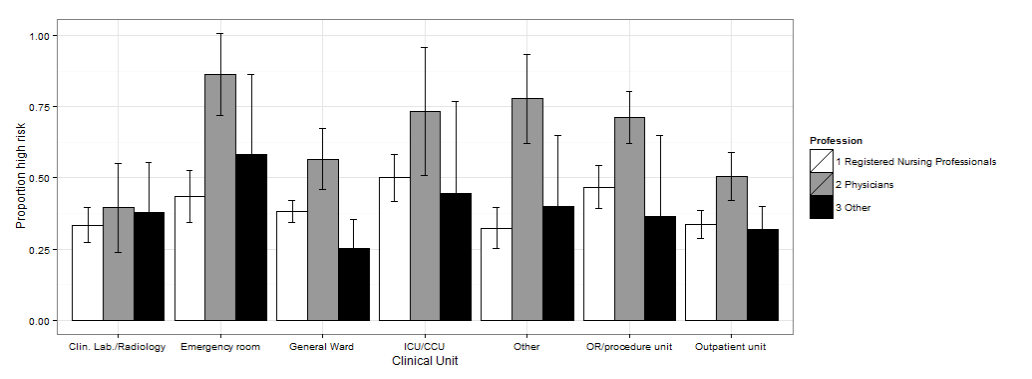
Proportion of high risk according to respondents’ professions and clinical unit (+95% CIs) in system-to-system interface errors.

**Figure 4:**
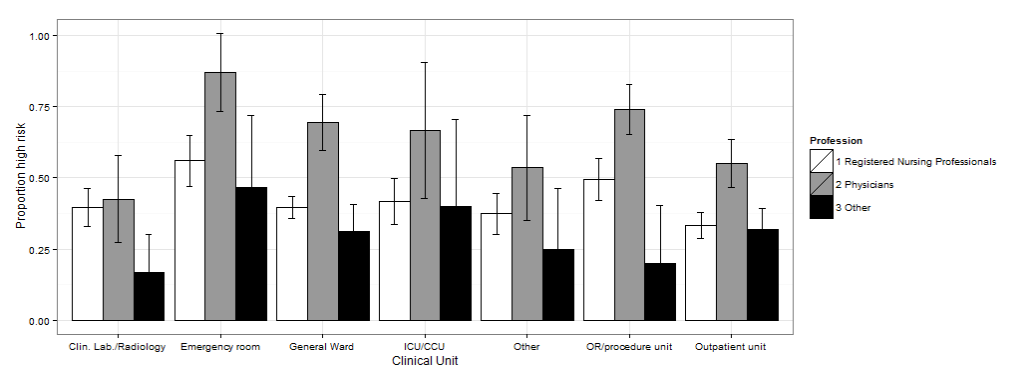
Proportion of high risk according to respondents’ professions and clinical unit (+95% CIs) in failure to find or use the most recent patient data.

In EDs and ORs we detected a general tendency towards relatively high-risk factors in all professional groups, except for *system interface errors* and *failures to find most recent patient information*, for which physicians reported higher risk levels than did nurses. Physicians generally tended to report higher risk for outpatient wards and general wards. Figures 1-4 show the proportion of high-risk assessments according to respondents’ professions and clinical units.

## Discussion

### Principal Results and Comparison with Prior Work

Research interest in EHR safety has been growing recently [25,26], but data specifically relating to EHR risk levels and severe-risk problem areas remain scarce, and to date no studies have explored this kind of specialized hospital context. One previous survey of risk managers and health care system lawyers provided valuable data about EHR-related serious events, but lacked EHR users’ perceptions. This previous survey also notes that additional data are needed to identify the extent of EHR-related safety concerns. To date, serious EHR-related events appear to be underreported and understudied [27].

Our study findings are based on a large number of EHR users in hospitals with a 100% degree of EHR implementation; approximately 92% of respondents used the EHR system several times per shift. Consequently, respondents were well aware of existing EHR safety concerns in their working environment. Despite the lack of similar studies, our results can be compared with previous study results.

Almost half of the respondents reported a severe perceived risk level related to *extended EHR unavailability*, which was perceived to be an especially high-risk area in EDs and CCUs. Although previous studies have not found this result, it can be explained by the fact that the literature has recognized *error type* as a high priority practice in all areas of EHR safety and, as such, a critical safety issue. Loss of continuous access to patient information risks leading to patient injuries [28]. Our finding of severe perceived risk can also be explained by hospitals with 100% EHR adoption rates, where paper records are no longer in use and comprehensive contingency plans have seen only partial implementation. Our results stress the importance of contingency planning, which includes processes and preparations that should be available when an incident occurs. The organizations’ activities, structured processes, and tasks are core requirements to continue operating and to minimize patient risk [29-32]. This area is important, especially because unexpected downtimes related to EHRs are fairly common in US-based health care organizations [33], and also occurred in this study. Moreover, this EHR concern merits greater interest, as the adoption of EHR systems has grown in recent years and continues to grow steadily [34]. A recent study in the United States shows that concerns about future EHR-use are related to the prolonged downtime of EHR systems, even if such incidents have seldom occurred in the past five years [27]. The potential consequences of an EHR downtime failure have become a cause for increasing concern as hospitals and health care organizations adopt large-scale EHR systems to handle many operations within the broader health care system. This also means that downtime can quickly affect not just a single ward or department, but an entire community [2,34,35]. We seek to emphasize how potential risks related to EHR downtimes are known to occur long after implementation [2]. Our study reinforces this previous result.

Previous studies have also shown that most (94%) safety concerns are related to either unmet data-display needs in the EHR, software upgrades or modifications, data transmission between components of the EHR, or hidden dependencies within the EHR [28]. In our study, approximately 40% of severe perceived risk was related to *system-to-system interface errors*, failure to find or use the most recent data, *EHR time measurement errors*, and *open or incomplete orders*. Unlike previously published studies, the lowest overall risk level in this study was associated with *selecting an incorrect item from a list of items*. *Selecting an incorrect item from a list of items* is partly a user interface issue, and previous studies have shown that usability is a key attribute of EHR system quality among users [32,36]. Studies have also reported that clinicians’ safety concerns often stem from EHR design and usability which fail to meet user requirements [37]. Our result for this specific error type may result from regulations [38] related to the safety and performance of medical devices in the EU. Products that fall within this scope (eg medical software) must meet all applicable essential safety requirements and must bear an EC conformity mark to indicate that they comply with all relevant EU directives. Manufacturers may only put medical devices into service that do not compromise the safety and health of patients, users and others. Therefore, the most obvious issues in the program (eg overly narrow columns in the drop-down menus) have been corrected.

In this study, profession proved to be a strong predictor for severe perceived risk, alongside clinical unit. Physicians reported a higher perceived risk with all EHR problem areas and factors. Large questionnaire studies in Finland have explored physicians’ views about EHR development and confirmed that physicians were critical of their IT systems [39]. High satisfaction among physicians associated strongly with perceived benefits [40]. In Finland, the previous survey results [39] showed that the EHR tools that physicians used daily can lead to a waste of operative resources and hinder physicians’ work. This result may also partly explain the physicians’ perceptions in this study, but this question requires further research.

In EDs, ORs, and to a somewhat lesser degree ICUs, the risk factors tended to be relatively high for all professional groups, except for *system interface errors* and *failures to locate the most recent patient information*, for which physicians reported higher risk levels than did nurses. A recent study indicates that the use of EHR technology strongly impacts ICU physician work (eg more time spent on clinical review and documentation) and workflow (eg clinical review and documentation becoming the focal point of many other tasks) [41]. Studies in the literature have examined the unintended consequences of information systems in EDs. The unique and particularly challenging characteristics of EDs, including rapid turnover, frequent transitions in care, constant interruptions, variation in patient volumes, and unfamiliar patients, make the ED environment particularly prone to errors. Thus, those implementing and maintaining HIT in such environments must give these factors careful consideration [42].

Participation in eLearning courses on EHR-use was associated with lower risk for some of the risk factors. Conversely, self-reported poor EHR-use skills were associated with higher risk scoring. This result can be viewed in the light of previous research. One of the major factors limiting clinicians’ adoption of an EHR system is low computer literacy and inadequate EHR training. A general consensus suggests a need for on-going support and additional systems training to optimize the efficient use of EHRs, but studies in this area are few. One study often identified learning as a necessary and inevitable condition for the efficient use of EHR [43,44]. Training supports EHR adoption and use, and according Ventres, high-quality training improves physicians’ proficiency in using an EHR system [45]. Consistent with these results, inadequate and poor-quality training was associated with poor utilization of EHR and participants failed to benefit from the full potential of the EHR system [46] *.* Additionally, one should take into account the broader educational perspective of informatics when striving to achieve safe care; informatics is an essential component of health care organizations’ skills and HIT safety, and should be integrated into educational programs [47,48]. Consequently, EHR training and skills supporting more efficient use seem to affect how EHR safety issues are controlled. Thus, EHR training is one core solution for meeting EHR safety concerns resulting from the failure to use HIT appropriately, or the misuse of HIT.

Finally, because comprehensive data on IT-related safety events are lacking, alternative approaches are needed to assess and respond appropriately to the HIT-related safety risks. The health information technology safety (HITS) framework described in a recent paper suggests that organizations will change their existing patient safety structures and processes to incorporate the unique set of skills needed for comprehensive HITS measurement. Organizations are encouraged to use clinicians trained in clinical informatics, and utilize a multidisciplinary oversight committee to help identify and prioritize risks [49]. The questionnaire developed for this study is one potential tool for this kind of approach.

### Limitations

Readers should take into account certain limitations of our study. Like all questionnaire studies, ours was subject to potential problems associated with response bias [50,51]. Some employees who responded to our survey may have had a greater interest in problems with EHRs than did non-responders. Thus, although our data may overestimate the actual risk level of electronic health records, it still provides valuable new information, especially about the variables associated with the most critical problem areas.

Possible validity and reliability weaknesses of the questionnaire are the most significant issues to be taken into account in this type of research. Considerable resources served to ensure a process of translation and adaptation in this study. The multi-phased questionnaire development process aimed to ensure semantic equivalence of the translated terms, thereby rendering good final face validity.

Some limitations in the study design limit one’s capacity to generalize the findings to a wider context. The response rate was relatively low, as is typical of many questionnaire studies [51,52]. Time constraints are reportedly a major barrier to studying health care professionals’ perceptions in this hospital setting. Consideration of the length of the questionnaire is thus relevant. Our questionnaire is designed to address the most important EHR problem areas at this time, and shortening it would have proved difficult. In the future, however, these problem areas may be revised as needed.

The use of qualitative assessment scales is subjective, and raters tend to vary. The fact that the personnel at responding hospitals systematically received training in the use of the risk matrix as part of the patient safety program significantly increased the reliability of this study.

### Conclusions

In conclusion, HIT safety hazards should be taken very seriously. Health care organizations should systematically assess EHR risks before harmful events occur. On the basis of this questionnaire study of 2864 respondents, our study indicates that the error type *extended EHR unavailability* is perceived as the most serious safety concern. The perceived risk ratings were relatively high for all professional groups in EDs and ORs. Consequently, implementing and maintaining EHRs in these areas will require consideration and follow-up.

Previous participation in eLearning courses on EHR-use was associated with lower risk for some of the risk factors. EHR training programs and preferably well-designed eTraining courses should be compulsory for all EHR users. EHR training is an important solution in meeting EHR safety concerns resulting from the failure to use HIT appropriately.

## Appendix

Multimedia Appendix 1 Sum variable means and reliability estimates (N=2678).

Multimedia Appendix 2 Distribution of background variables and proportion of respondents reporting a high- or extreme-risk severity rating, including margins of error and 95 % CIs for the proportions (N=2864).

Multimedia Appendix 3 Association of background variables with perceived EHR risk rating (Odds ratios [OR], 95 % CIs, and P-values [P] from logistic regression analyses).

Multimedia Appendix 4 Table A3. Proportion of high risk according to respondents’ professions and clinical unit (+95% confidence intervals) by risk type including margin of errors and 95 % confidence intervals for the proportions (N=2,864).

Multimedia Appendix 5 Translation of Questionnaire Items from Finnish to English.

## Abbreviations

CCU: critical care unit
ED: emergency department
EHR: electronic health record
EU: European Union
HFMEA: health care failure mode and effects analysis
HIS: health information system
HIT: health information technology
HITS: health information technology safety
HR: human resources
ICU: intensive care unit
IT: information technology
OR: operating room
