# Supplementary material for: Electronic Health Record-Related Safety Concerns: A Cross-Sectional Survey of Electronic Health Record Users
Source: JMIR Med Inform. 2016 May 6;4(2):e13. doi: 10.2196/medinform.5238 (PMC4890731; doi:10.2196/medinform.5238)
Supplement: Multimedia Appendix 1 [file medinform_v4i2e13_app1.pdf]

Appendix 1:Table A1. Sum variable means and reliability estimates (N=2,678)

Variable	Mean of sum variable[a]	SE[b] of sum variable	Cronbach's alpha	[95% CI[c]]	Complete cases[d]	% Complete cases
Incorrect patient identification	2.38	1.05	.858	[0.847 - 0.868]	1225	42.7
Extended EHR unavailability	2.82	1.04	.864	[0.856 - 0.873]	996	34.7
Failure to heed a computer-generated warning or alert	2.28	0.92	.866	[0.856 - 0.875]	983	34.3
System-to-system interface errors	2.59	1.08	.880	[0.872 - 0.888]	983	34.3
Failure to find or use the most recent patient data	2.58	0.95	.868	[0.859 - 0.877]	1546	53.9
EHR time measurement translational challenges	2.58	1.03	.870	[0.860 - 0.879]	877	30.6
Incorrect item selected from a list of items	2.36	0.99	.888	[0.879 - 0.896]	1218	42.5
Open, incomplete or missing orders	2.74	1.02	.789	[0.773 - 0.803]	1389	48.4

[a]Question items were measured on a scale of 1 to 5

[b]SE=Standard Error

[c]CI=Confidence Interval

[d] Respondents who answered every single item in the multi-item scale (between 3 to 6 items/scale)
