# Supplementary material for: Electronic Health Record-Related Safety Concerns: A Cross-Sectional Survey of Electronic Health Record Users
Source: JMIR Med Inform. 2016 May 6;4(2):e13. doi: 10.2196/medinform.5238 (PMC4890731; doi:10.2196/medinform.5238)
Supplement: Multimedia Appendix 2 [file medinform_v4i2e13_app2.pdf]

Appendix 2: Table A2. Distribution of background variables and proportion of respondents reporting a high or extreme risk severity rating including margin of errors and 95 % confidence intervals for the proportions (N=2,864).

			**Percent of all respondents reporting a high risk level (in %)**											
			**Incorrect patient identification**			**Extended EHR unavailability**			**Failure to heed a computer-generated warning or alert**			**System-to-system interface errors**		
**Variable**	N	%	%	Margin of Error	95 % CI	%	Margin of Error	95 % CI	%	Margin of Error	95 % CI	%	Margin of Error	95 % CI
**Gender**														
Female	2439	85,2	31,6	1,85	29.8-33.5	49,0	1,98	47.0-51.0	30,0	1,82	28.2-31.9	40,4	1,95	38.5-42.4
Male	425	14,8	31,6	4,42	27.2-36.0	49,0	4,75	44.3-53.8	34,2	4,51	29.7-38.7	46,1	4,74	41.4-50.9
**Age Group**														
20-34	637	22,2	30,7	3,58	27.1-34.3	47,4	3,88	43.5-51.3	28,2	3,49	24.7-31.7	42,1	3,83	38.3-45.9
35-49	1123	39,2	30,1	2,68	27.4-32.7	48,5	2,92	45.6-51.4	31,1	2,71	28.4-33.9	41,7	2,88	38.8-44.6
50-59	851	29,7	31,7	3,13	28.6-34.8	49,5	3,36	46.1-52.9	30,9	3,11	27.8-34	39,9	3,29	36.6-43.2
60+	252	8,8	41,2	6,08	35.1-47.3	53,4	6,16	47.3-59.6	34,4	5,87	28.5-40.3	42,2	6,10	36.1-48.3
**Professional Qualification**														
Registered Nurses	2044	71,4	31,9	2,02	29.8-33.9	47,9	2,17	45.8-50.1	28,4	1,96	26.5-30.4	38,3	2,11	36.2-40.4
Physicians	433	15,1	36,2	4,53	31.7-40.7	65,2	4,49	60.7-69.7	45,2	4,69	40.5-49.8	59,9	4,62	55.3-64.5
Clinical Clerks	246	8,6	28,2	5,63	22.6-33.9	40,4	6,13	34.3-46.6	22,9	5,25	17.6-28.1	35,4	5,98	29.4-41.4
Academic specialists	141	4,9	17,4	6,26	11.2-23.7	26,4	7,27	19.1-33.6	22,0	6,84	15.1-28.8	29,0	7,49	21.5-36.5
**Education**														
RN or other polytechnic degree	1627	56,8	31,5	2,26	29.3-33.8	47,4	2,43	44.9-49.8	27,8	2,18	25.7-30	37,7	2,36	35.4-40.1
Vocational Training	394	13,8	30,8	4,56	26.3-35.4	44,7	4,91	39.7-49.6	25,3	4,29	21-29.5	33,9	4,67	29.2-38.6
Other Tertiary Education	142	5,0	38,9	8,02	30.9-47.0	52,2	8,22	44.0-60.4	34,5	7,82	26.7-42.3	46,7	8,21	38.5-54.9
Master's Degree/ MD	437	15,3	28,4	4,23	24.1-32.6	55,0	4,66	50.3-59.7	40,8	4,61	36.2-45.4	54,1	4,67	49.5-58.8
PhD	188	6,6	36,7	6,89	29.8-43.6	59,0	7,03	52.0-66.0	39,1	6,97	32.1-46	54,7	7,12	47.6-61.8
Other	76	2,7	30,2	10,32	19.8-40.5	38,6	10,94	27.6-49.5	24,6	9,68	14.9-34.2	30,5	10,35	20.2-40.9
**Clinical Unit**														
Clinical Laboratory/Radiology	334	11,7	35,3	5,12	30.2-40.4	38,5	5,22	33.2-43.7	16,9	4,02	12.9-20.9	34,7	5,11	29.6-39.8
Emergency room	159	5,6	44,8	7,73	37.1-52.5	58,8	7,65	51.2-66.5	39,3	7,59	31.7-46.9	51,0	7,77	43.3-58.8
General Ward	883	30,8	29,4	3,01	26.4-32.4	49,9	3,30	46.6-53.2	32,5	3,09	29.4-35.6	39,1	3,22	35.9-42.3
ICU/CCU	176	6,1	27,7	6,61	21.1-34.3	51,2	7,38	43.8-58.6	26,1	6,48	19.6-32.5	51,8	7,38	44.4-59.2
Labor ward	73	2,5	22,2	9,54	12.7-31.8	45,1	11,41	33.7-56.5	31,3	10,64	20.7-42	35,8	11,00	24.8-46.8
OR or procedure unit	301	10,5	44,6	5,62	38.9-50.2	63,4	5,44	57.9-68.8	35,7	5,41	30.3-41.2	54,8	5,62	49.2-60.4
Outpatient unit	755	26,4	26,2	3,14	23.1-29.4	46,7	3,56	43.1-50.2	30,0	3,27	26.7-33.2	37,0	3,44	33.6-40.4
Other	183	6,4	30,9	6,69	24.2-37.6	38,8	7,06	31.7-45.8	30,6	6,68	24-37.3	40,3	7,11	33.2-47.4

Variable	N	%	Percent of all respondents reporting a high risk level (in %): Incorrect patient identification %	Margin of Error	95 % CI	Extended EHR unavailability %	Margin of Error	95 % CI	Failure to heed a computer-generated warning or alert %	Margin of Error	95 % CI	System-to-system interface errors %	Margin of Error	95 % CI
**Work Experience**														
0-5 years	614	21,4	28,8	3,58	25.2-32.4	44,2	3,93	40.3-48.1	27,0	3,51	23.5-30.5	40,2	3,88	36.3-44.1
6-15 years	974	34,0	31,2	2,91	28.3-34.1	49,2	3,14	46.1-52.4	32,0	2,93	29.1-34.9	42,0	3,10	38.9-45.1
16-25 years	659	23,0	29,6	3,49	26.1-33.1	51,1	3,82	47.3-54.9	31,6	3,55	28-35.1	42,5	3,77	38.7-46.3
25+ years	617	21,5	37,6	3,82	33.8-41.4	51,0	3,94	47.1-55.0	31,4	3,66	27.7-35	40,2	3,87	36.3-44.1
**Frequency of EHR Use**														
Several times per shift	2640	92,2	31,8	1,78	30.1-33.6	49,4	1,91	47.5-51.3	30,5	1,76	28.7-32.2	41,6	1,88	39.7-43.5
Less often	224	7,8	29,1	5,95	23.2-35.1	43,5	6,49	37.0-49.9	33,5	6,18	27.4-39.7	38,1	6,36	31.7-44.4
**EHR Training Mode**														
Classroom	610	21,3	29,0	3,60	25.4-32.6	49,5	3,97	45.5-53.4	32,4	3,71	28.7-36.1	40,7	3,90	36.8-44.6
eLearning	880	30,7	28,1	2,97	25.2-31.1	44,8	3,29	41.6-48.1	26,2	2,90	23.3-29.1	38,3	3,21	35.1-41.5
General	803	28,0	34,8	3,29	31.5-38.1	51,5	3,46	48.0-54.9	32,2	3,23	29-35.5	43,0	3,42	39.5-46.4
IT Support	304	10,6	34,3	5,34	28.9-39.6	49,0	5,62	43.4-54.6	30,8	5,19	25.6-36	41,8	5,54	36.2-47.3
Other/no training	267	9,3	37,0	5,79	31.2-42.8	54,1	5,98	48.1-60.1	38,1	5,82	32.3-43.9	47,9	5,99	41.9-53.9
**EHR Skills Level**														
Good	793	27,7	28,9	3,15	25.7-32.0	49,1	3,48	45.6-52.6	28,4	3,14	25.2-31.5	40,3	3,41	36.9-43.7
Fair	1590	55,5	30,6	2,26	28.3-32.8	47,9	2,46	45.4-50.3	29,1	2,23	26.8-31.3	40,4	2,41	38-42.8
Poor	481	16,8	40,0	4,38	35.7-44.4	52,6	4,46	48.2-57.1	39,8	4,38	35.5-44.2	46,4	4,46	41.9-50.9
**Total**	**2864**	**100,0**	**31,6**	**1,70**	**29.9-33.3**	**49,0**	**1,83**	**47.1-50.8**	**30,7**	**1,69**	**29.0-32.4**	**41,3**	**1,80**	**39.5-43.1**

Variable	N	%	Percent of all respondents reporting a high risk level (in %): Failure to find or use the most recent patient data %	Margin of Error	95 % CI	EHR time measurement translational challenges %	Margin of Error	95 % CI	Incorrect item selected from a list of items %	Margin of Error	95 % CI	Open, incomplete or missing orders %	Margin of Error	95 % CI
**Gender**														
Female	2439	85,2	41,5	1,96	39.6-43.5	35,7	1,90	33.8-37.6	26,7	1,76	24.9-28.4	40,3	1,95	38.3-42.2
Male	425	14,8	48,7	4,75	44.0-53.5	40,9	4,67	36.2-45.5	33,3	4,48	28.9-37.8	42,5	4,70	37.8-47.2
**Age Group**														
20-34	637	22,2	41,8	3,83	38.0-45.7	33,4	3,66	29.7-37.1	25,7	3,39	22.3-29.1	40,6	3,81	36.8-44.5
35-49	1123	39,2	43,0	2,90	40.1-45.9	37,9	2,84	35.0-40.7	27,5	2,61	24.9-30.1	43,5	2,90	40.6-46.4
50-59	851	29,7	42,7	3,32	39.4-46.0	36,9	3,24	33.6-40.1	29,9	3,07	26.8-32.9	38,6	3,27	35.3-41.9
60+	252	8,8	42,4	6,10	36.3-48.5	37,9	5,99	31.9-43.9	27,7	5,52	22.1-33.2	33,7	5,84	27.9-39.5

Variable	N	%	Percent of all respondents reporting a high risk level (in %)											
			Failure to find or use the most recent patient data			EHR time measurement translational challenges			Incorrect item selected from a list of items			Open, incomplete or missing orders		
			%	Margin of Error	95 % CI	%	Margin of Error	95 % CI	%	Margin of Error	95 % CI	%	Margin of Error	95 % CI
**Professional Qualification**														
Registered Nurses	2044	71,4	40,1	2,13	38.0-42.3	35,8	2,08	33.7-37.9	25,4	1,89	23.5-27.3	40,4	2,13	38.3-42.6
Physicians	433	15,1	63,1	4,54	58.6-67.7	47,8	4,70	43.1-52.5	41,7	4,64	37.0-46.3	48,0	4,71	43.3-52.7
Clinical Clerks	246	8,6	30,7	5,76	24.9-36.5	22,0	5,18	16.8-27.2	23,9	5,33	18.6-29.2	37,4	6,05	31.4-43.5
Academic specialists	141	4,9	29,8	7,55	22.3-37.4	20,3	6,64	13.7-26.9	17,5	6,28	11.3-23.8	18,2	6,37	11.8-24.5
**Education**														
RN or other polytechnic degree	1627	56,8	40,1	2,38	37.7-42.4	35,0	2,32	32.6-37.3	25,1	2,11	23.0-27.3	41,0	2,39	38.6-43.4
Vocational Training	394	13,8	31,5	4,59	26.9-36.1	31,3	4,58	26.7-35.8	23,5	4,19	19.3-27.7	33,3	4,65	28.7-38.0
Other Tertiary Education	142	5,0	46,2	8,20	38.0-54.4	40,0	8,06	31.9-48.1	31,6	7,65	24.0-39.3	43,4	8,15	35.2-51.5
Master's Degree/ MD	437	15,3	55,3	4,66	50.6-59.9	44,1	4,65	39.4-48.7	34,7	4,46	30.2-39.2	41,2	4,61	36.5-45.8
PhD	188	6,6	53,9	7,13	46.8-61.0	41,1	7,03	34.0-48.1	40,1	7,01	33.1-47.1	47,6	7,14	40.5-54.7
Other	76	2,7	43,3	11,14	32.1-54.4	30,2	10,32	19.9-40.5	21,7	9,26	12.4-30.9	41,9	11,09	30.8-53.0
**Clinical Unit**														
Clinical Laboratory/Radiology	334	11,7	37,6	5,19	32.4-42.8	29,1	4,87	24.2-34.0	22,1	4,45	17.6-26.5	22,6	4,49	18.1-27.1
Emergency room	159	5,6	59,7	7,62	52.1-67.4	52,2	7,76	44.4-60.0	34,7	7,40	27.3-42.1	62,9	7,51	55.4-70.4
General Ward	883	30,8	41,8	3,25	38.5-45.0	35,0	3,15	31.9-38.2	27,7	2,95	24.8-30.7	41,6	3,25	38.4-44.9
ICU/CCU	176	6,1	44,0	7,33	36.6-51.3	40,5	7,25	33.3-47.8	28,8	6,69	22.1-35.4	44,3	7,34	37.0-51.6
Labor ward	73	2,5	38,0	11,14	26.9-49.2	23,6	9,75	13.9-33.4	16,7	8,55	8.1-25.2	35,3	10,96	24.3-46.3
OR or procedure unit	301	10,5	56,2	5,61	50.6-61.8	51,3	5,65	45.6-56.9	37,9	5,48	32.4-43.4	52,8	5,64	47.2-58.5
Outpatient unit	755	26,4	37,2	3,45	33.8-40.7	31,3	3,31	28.0-34.6	25,4	3,10	22.3-28.5	33,4	3,37	30.1-36.8
Other	183	6,4	38,9	7,06	31.8-46.0	37,8	7,03	30.8-44.9	25,8	6,34	19.4-32.1	37,7	7,02	30.7-44.7
**Work Experience**														
0-5 years	614	21,4	36,8	3,82	33.0-40.6	30,2	3,63	26.5-33.8	22,6	3,31	19.3-25.9	38,5	3,85	34.6-42.3
6-15 years	974	34,0	44,0	3,12	40.9-47.1	38,0	3,05	34.9-41.0	28,8	2,85	26.0-31.7	42,9	3,11	39.8-46.0
16-25 years	659	23,0	44,5	3,79	40.7-48.3	40,8	3,75	37.1-44.6	28,3	3,44	24.8-31.7	41,8	3,77	38.1-45.6
25+ years	617	21,5	44,2	3,92	40.3-48.1	36,2	3,79	32.4-40.0	30,9	3,64	27.2-34.5	37,6	3,82	33.8-41.4
**Frequency of EHR Use**														
Several times per shift	2640	92,2	43,0	1,89	41.1-44.9	36,7	1,84	34.9-38.6	27,7	1,71	26.0-29.4	40,7	1,87	38.8-42.6
Less often	224	7,8	37,0	6,32	30.7-43.3	34,3	6,22	28.1-40.5	28,5	5,91	22.6-34.4	39,7	6,41	33.3-46.1

Variable	N	%	Percent of all respondents reporting a high risk level (in %) Failure to find or use the most recent patient data %	Margin of Error	95 % CI	EHR time measurement translational challenges %	Margin of Error	95 % CI	Incorrect item selected from a list of items %	Margin of Error	95 % CI	Open, incomplete or missing orders %	Margin of Error	95 % CI
**EHR Training Mode**														
Classroom	610	21,3	43,4	3,93	39.5-47.4	37,7	3,85	33.8-41.5	25,3	3,45	21.9-28.8	39,3	3,88	35.4-43.1
eLearning	880	30,7	38,3	3,21	35.1-41.5	33,8	3,13	30.7-36.9	25,4	2,88	22.5-28.3	38,2	3,21	35.0-41.4
General	803	28,0	43,7	3,43	40.3-47.2	37,7	3,35	34.3-41.0	29,9	3,17	26.7-33.0	44,6	3,44	41.1-48.0
IT Support	304	10,6	46,0	5,60	40.4-51.6	35,9	5,39	30.5-41.3	28,4	5,07	23.3-33.4	38,1	5,46	32.7-43.6
Other/no training	267	9,3	48,4	5,99	42.4-54.4	41,0	5,90	35.1-46.9	35,0	5,72	29.3-40.7	43,7	5,95	37.8-49.7
**EHR Skills Level**														
Good	793	27,7	39,0	3,39	35.6-42.4	36,5	3,35	33.1-39.8	25,6	3,04	22.6-28.7	43,7	3,45	40.3-47.2
Fair	1590	55,5	42,9	2,43	40.4-45.3	34,8	2,34	32.4-37.1	27,6	2,20	25.4-29.8	38,1	2,39	35.7-40.5
Poor	481	16,8	48,1	4,47	43.6-52.6	43,0	4,42	38.6-47.4	31,6	4,16	27.5-35.8	44,2	4,44	39.8-48.7
**Total**	**2864**	**100,0**	**42,6**	**1,81**	**40.8-44.4**	**36,6**	**1,76**	**34.8-38.3**	**27,7**	**1,64**	**26.1-29.4**	**40,6**	**1,80**	**38.8-42.4**
