# Supplementary material for: Electronic Health Record-Related Safety Concerns: A Cross-Sectional Survey of Electronic Health Record Users
Source: JMIR Med Inform. 2016 May 6;4(2):e13. doi: 10.2196/medinform.5238 (PMC4890731; doi:10.2196/medinform.5238)
Supplement: Multimedia Appendix 3 [file medinform_v4i2e13_app3.pdf]

Table 2

	Incorrect patient identification			Extended EHR unavailability			Failure to heed a computer-generated warning or alert		
	OR	95% CI	P	OR	95% CI	P	OR	95% CI	P
Intercept	0,31	0.23 - 0.42	***<.001***	0,92	0.69 - 1.21	0,547	0,39	0.28 - 0.54	**<.001**
**Profession**									
Registered Nurses	1.00	-	-	1.00	-	-	1.00	-	-
Physicians	1,11	0.88 - 1.40	0,376	1.90	1.52 - 2.38	**<.001**	2,03	1.60 - 2.57	**<.001**
Clinical Clerks	0,83	0.59 - 1.16	0,278	0,66	0.49 - 0.89	**0,006**	0,74	0.49 - 1.08	0,128
Academic specialists	0,48	0.28 - 0.78	**0,005**	0,41	0.27 - 0.61	**<.001**	0,71	0.41 - 1.17	0,195
**Clinical Unit**									
General Ward	1.00	-	-	1.00	-	-	1.00	-	-
Clin. Lab./Radiology	1,21	0.91 - 1.62	0,187	0,57	0.43 - 0.75	**<.001**	0,38	0.25 - 0.56	**<.001**
Emergency room	1,93	1.35 - 2.76	**<.001**	1,39	0.98 - 1.99	0,068	1.30	0.89 - 1.89	0,170
ICU/CCU	0,91	0.61 - 1.33	0,626	1,02	0.73 - 1.43	0,906	0,73	0.48 - 1.10	0,139
Labor ward	0.70	0.38 - 1.23	0,234	0,82	0.50 - 1.34	0,424	0,97	0.55 - 1.66	0,912
OR or procedure unit	1,79	1.34 - 2.38	**<.001**	1,44	1.09 - 1.91	**0,011**	0,93	0.68 - 1.27	0,650
Outpatient unit	0,85	0.67 - 1.08	0,194	0,87	0.70 - 1.07	0,176	0,84	0.66 - 1.07	0,170
Other	1,04	0.70 - 1.52	0,851	0,59	0.42 - 0.84	**0,003**	0,87	0.57 - 1.32	0,530
**Professional Experience**									
0-5 years	1.00	-	-	1.00	-	-	1.00	-	-
6-15 years	1.10	0.87 - 1.40	0,420	1,13	0.91 - 1.40	0,275	1,18	0.91 - 1.52	0,216
16-25 years	1,02	0.79 - 1.33	0,853	1,24	0.98 - 1.57	0,072	1,15	0.87 - 1.53	0,312
25+ years	1,42	1.09 - 1.84	**0,009**	1,32	1.04 - 1.67	**0,024**	1,17	0.87 - 1.56	0,302
**EHR training**									
Class Room Training	1.00	-	-	1.00	-		1.00	-	-
eLearning	0,93	0.73 - 1.19	0,557	0,78	0.63 - 0.98	**0,030**	0,73	0.56 - 0.94	**0,015**
General	1,31	1.03 - 1.69	**0,031**	1,15	0.92 - 1.44	0,230	1,01	0.78 - 1.32	0,929
IT Support	1,25	0.91 - 1.71	0,165	1.00	0.75 - 1.34	0,987	1.00	0.71 - 1.40	0,996
Other/no training	1,29	0.92 - 1.81	0,133	1,28	0.93 - 1.76	0,126	1,37	0.95 - 1.98	0,092
**EHR skills**									
Good	1.00	-	-	1.00	-	-	1.00	-	-
Fair	1,07	0.87 - 1.31	0,515	0,93	0.77 - 1.11	0,429	1,03	0.83 - 1.28	0,786
Poor	1,56	1.20 - 2.03	***0,001***	1,04	0.81 - 1.33	0,752	1,58	1.19 - 2.08	**0,001**
Observations		2598			2732			2291	

	System-to-system interface errors			Failure to find or use the most recent patient data			EHR time measurement translational challenges		
	OR	95% CI	P	OR	95% CI	P	OR	95% CI	P
Intercept	0,57	0.42 - 0.77	**<.001**	0,52	0.39 - 0.69	**<.001**	0,47	0.34 - 0.65	**<.001**
**Profession**									
Nurses	1.00	-	-	1.00	-	-	1.00	-	-
Physicians	2,37	1.89 - 2.98	**<.001**	2,51	2.00 - 3.15	**<.001**	1,51	1.19 - 1.91	**0,001**
Clinical Clerks	0.90	0.64 - 1.27	0,565	0,69	0.49 - 0.95	**0,023**	0,51	0.32 - 0.77	**0,002**
Academic specialists	0,75	0.47 - 1.17	0,216	0,72	0.46 - 1.09	0,123	0,51	0.27 - 0.92	**0,032**
**Clinical Unit**									
General Ward	1.00	-	-	1.00	-	-	1.00	-	-
Clin. Lab./Radiology	0,77	0.57 - 1.03	0,083	0,76	0.57 - 1.02	0,072	0,73	0.53 - 1.01	0,059
Emergency room	1,57	1.09 - 2.25	**0,015**	2,06	1.44 - 2.96	**<.001**	1,98	1.36 - 2.89	**<.001**
ICU/CCU	1,69	1.20 - 2.39	**0,003**	1,07	0.76 - 1.51	0,691	1,23	0.85 - 1.76	0,264
Labor ward	0,87	0.51 - 1.47	0,613	0,86	0.51 - 1.42	0,567	0,56	0.28 - 1.04	0,079
OR or procedure unit	1,53	1.14 - 2.04	**0,004**	1,42	1.07 - 1.89	**0,017**	1,67	1.22 - 2.28	**0,001**

	OR	95% CI	P	OR	95% CI	P	OR	95% CI	P
Outpatient unit	0,84	0.67 - 1.05	0,127	0,77	0.62 - 0.96	**0,018**	0,82	0.64 - 1.06	0,131
Other	1.00	0.68 - 1.45	0,981	0,82	0.56 - 1.19	0,295	1.10	0.72 - 1.67	0,655
**Professional Experience**									
0-5 years	1.00	-	-	1.00	-	-	1.00	-	-
6-15 years	0,98	0.78 - 1.23	0,853	1,25	1.00 - 1.56	0,053	1,32	1.03 - 1.70	**0,030**
16-25 years	1,01	0.79 - 1.29	0,938	1,31	1.03 - 1.67	**0,030**	1,49	1.14 - 1.97	**0,004**
25+ years	0,98	0.76 - 1.27	0,894	1,33	1.04 - 1.71	**0,025**	1,29	0.97 - 1.72	0,084
**EHR training**									
Class Room Training	1.00	-	-	1.00	-	-	1.00	-	-
eLearning	0,88	0.70 - 1.11	0,289	0,76	0.61 - 0.95	**0,018**	0,81	0.63 - 1.05	0,110
General	1,08	0.84 - 1.37	0,554	1,04	0.82 - 1.31	0,770	1,04	0.79 - 1.35	0,796
IT Support	1,05	0.77 - 1.43	0,76	1,12	0.83 - 1.50	0,459	0,92	0.66 - 1.28	0,613
Other/no training	1.30	0.93 - 1.82	0,122	1,17	0.84 - 1.62	0,342	1,12	0.78 - 1.59	0,539
**EHR skills**									
Good	1.00	-	-	1.00	-	-	1.00	-	-
Fair	1,01	0.83 - 1.22	0,954	1,15	0.96 - 1.40	0,138	0,89	0.72 - 1.10	0,270
Poor	1,27	0.98 - 1.64	0,073	1,35	1.05 - 1.74	**0,021**	1,21	0.91 - 1.60	0,183
Observations		2460			2618			2158	

	Incorrect item selected from a list of items			Open, incomplete or missing orders		
	OR	95% CI	P	OR	95% CI	P
Intercept	0,23	0.16 - 0.33	**<.001**	0,74	0.55 - 1.00	0,054
**Profession**						
Nurses	1.00	-	-	1.00	-	-
Physicians	1,99	1.57 - 2.52	**<.001**	1.30	1.03 - 1.65	**0,024**
Clinical Clerks	0,95	0.65 - 1.39	0,813	0,77	0.55 - 1.06	0,114
Academic specialists	0,69	0.39 - 1.16	0,184	0,38	0.21 - 0.66	**0,001**
**Clinical Unit**						
General Ward	1.00	-	-	1.00	-	-
Clin. Lab./Radiology	0,66	0.46 - 0.94	**0,024**	0,39	0.25 - 0.58	**<.001**
Emergency room	1,35	0.92 - 1.98	0,123	2,27	1.59 - 3.28	**<.001**
ICU/CCU	1,05	0.70 - 1.54	0,816	1,09	0.77 - 1.54	0,632
Labor ward	0,53	0.26 - 1.00	0,063	0,75	0.44 - 1.25	0,281
OR or procedure unit	1,27	0.93 - 1.73	0,134	1,37	1.03 - 1.83	**0,032**
Outpatient unit	0,83	0.65 - 1.07	0,152	0,69	0.55 - 0.86	**0,001**
Other	0,85	0.54 - 1.29	0,444	0,83	0.56 - 1.22	0,354
**Professional Experience**						
0-5 years	1.00	-	-	1.00	-	-
6-15 years	1.30	1.00 - 1.68	0,051	1,17	0.93 - 1.48	0,192
16-25 years	1,29	0.97 - 1.71	0,082	1,12	0.87 - 1.44	0,397
25+ years	1,52	1.14 - 2.04	**0,005**	0,98	0.75 - 1.29	0,906
**EHR training**						
Class Room Training	1.00	-	-	1.00	-	-
eLearning	1.00	0.77 - 1.30	0,982	0,91	0.72 - 1.15	0,426
General	1,24	0.95 - 1.63	0,12	1,32	1.03 - 1.69	**0,028**
IT Support	1,22	0.87 - 1.72	0,25	0,92	0.66 - 1.27	0,613
Other/no training	1,58	1.10 - 2.28	**0,014**	1,15	0.81 - 1.65	0,434
**EHR skills**						
Good	1.00	-	-	1.00	-	-
Fair	1,09	0.88 - 1.36	0,446	0,79	0.64 - 0.96	**0,018**
Poor	1,25	0.94 - 1.67	0,121	1.00	0.76 - 1.31	0,999
Observations		2372			2345	
