# Supplementary material for: Electronic Health Record-Related Safety Concerns: A Cross-Sectional Survey of Electronic Health Record Users
Source: JMIR Med Inform. 2016 May 6;4(2):e13. doi: 10.2196/medinform.5238 (PMC4890731; doi:10.2196/medinform.5238)
Supplement: Multimedia Appendix 4 [file medinform_v4i2e13_app4.pdf]

Appendix 3: Table A3. Proportion of high risk according to respondents' professions and clinical unit (+95% confidence intervals) by risk type including margin of errors and 95 % confidence intervals for the proportions (N=2,864).

				**Percent of all respondents reporting a high risk level (in %)**											
				**Incorrect patient identification**			**Extended EHR unavailability**			**Failure to heed a computer-generated warning or alert**			**System-to-system interface errors**		
**Clinical Unit**	**Professional Qualification**	N	%	%	Margin of Error	95 % CI	%	Margin of Error	95 % CI	%	Margin of Error	95 % CI	%	Margin of Error	95 % CI
Clinical Lab /Radiology	Registered Nurses	254	8,9	39,8	6,02	33.8-45.9	39,3	6,01	33.3-45.3	17,9	4,72	13.2-22.7	33,5	5,80	27.7-39.3
	Physicians	42	1,5	22,5	12,63	9.9-35.1	37,5	14,64	22.9-52.1	16,7	11,27	5.4-27.9	39,5	14,78	24.7-54.3
	Other	38	1,3	18,2	12,26	5.9-30.4	33,3	14,99	18.3-48.3	9,5	9,33	0.2-18.9	37,9	15,43	22.5-53.4
Emergency	Registered Nurses	118	4,1	44,7	8,97	35.8-53.7	56,6	8,94	47.7-65.6	34,5	8,58	26.0-43.1	43,4	8,94	34.4-52.3
	Physicians	24	0,8	37,5	19,37	18.1-56.9	65,2	19,05	46.2-84.3	59,1	19,67	39.4-78.8	86,4	13,73	72.6-100.1
	Other	17	0,6	56,3	23,58	32.7-79.8	64,7	22,72	42.0-87.4	46,2	23,70	22.5-69.9	58,3	23,44	34.9-81.8
General Ward	Registered Nurses	704	24,6	30,4	3,40	27.0-33.8	49,2	3,69	45.5-52.9	31,0	3,42	27.6-34.5	38,3	3,59	34.7-41.9
	Physicians	85	3,0	28,4	9,59	18.8-38.0	71,8	9,57	62.2-81.3	53,7	10,60	43.1-64.3	56,6	10,54	46.1-67.2
	Other	94	3,3	22,4	8,42	13.9-30.8	34,4	9,61	24.8-44.1	18,8	7,89	10.9-26.6	25,4	8,79	16.6-34.1
ICU/CCU	Registered Nurses	150	5,2	28,1	7,20	21.0-35.3	50,3	8,00	42.3-58.4	24,4	6,87	17.5-31.2	50,0	8,00	42.0-58.0
	Physicians	15	0,5	21,4	20,76	0.7-42.2	64,3	24,25	40.0-88.5	30,8	23,36	7.4-54.1	73,3	22,38	51.0-95.7
	Other	11	0,4	30,0	27,08	2.9-57.1	45,5	29,43	16.0-74.9	40,0	28,95	11.0-69.0	44,4	29,36	15.1-73.8
OR/ procedure unit	Registered Nurses	186	6,5	42,9	7,11	35.7-50.0	59,3	7,06	52.3-66.4	30,5	6,62	23.9-37.1	46,7	7,17	39.5-53.9
	Physicians	98	3,4	47,3	9,88	37.4-57.2	74,0	8,69	65.3-82.6	46,4	9,87	36.5-56.3	71,3	8,96	62.3-80.2
	Other	17	0,6	47,1	23,73	23.3-70.8	47,1	23,73	23.3-70.8	16,7	17,72	-1-34.4	36,4	22,87	13.5-59.2
Outpatient unit	Registered Nurses	435	15,2	23,8	4,00	19.8-27.8	47,0	4,69	42.3-51.7	26,6	4,15	22.4-30.7	33,8	4,45	29.3-38.2
	Physicians	138	4,8	38,8	8,13	30.6-46.9	62,5	8,08	54.4-70.6	45,0	8,30	36.7-53.3	50,4	8,34	42.0-58.7
	Other	182	6,4	21,9	6,01	15.9-27.9	33,1	6,84	26.3-40.0	24,1	6,21	17.9-30.3	32,1	6,78	25.3-38.8
Other	Registered Nurses	197	6,9	28,0	6,27	21.7-34.3	38,3	6,79	31.5-45.0	30,0	6,40	23.6-36.4	32,3	6,53	25.8-38.8
	Physicians	31	1,1	35,7	16,87	18.8-52.6	67,7	16,46	51.3-84.2	42,9	17,42	25.4-60.3	77,8	14,63	63.1-92.4
	Other	28	1,0	16,7	13,80	2.9-30.5	22,7	15,52	7.2-38.2	15,4	13,36	2.0-28.7	40,0	18,15	21.9-58.1
**Total**	**Total**	**2864**	**100,0**	**31,6**	**1,70**	**29.9-33.3**	**49,0**	**1,83**	**47.1-50.8**	**30,7**	**1,69**	**29.0-32.4**	**41,3**	**1,80**	**39.5-43.1**

Clinical Unit	Professional Qualification	N	%	Percent of all respondents reporting a high risk level (in %) — Failure to find or use the most recent patient data: %	Margin of Error	95 % CI	EHR time measurement translational challenges: %	Margin of Error	95 % CI	Incorrect item selected from a list of items: %	Margin of Error	95 % CI	Open, incomplete or missing orders: %	Margin of Error	95 % CI
Clinical Labo /Radiology	Registered Nurses	254	8,9	39,7	6,02	33.7-45.7	29,3	5,60	23.7-34.9	21,0	5,01	16.0-26.0	26,2	5,41	20.8-31.6
	Physicians	42	1,5	42,5	14,95	27.5-57.5	25,7	13,22	12.5-38.9	34,3	14,36	19.9-48.6	10,7	9,35	1.4-20.1
	Other	38	1,3	16,7	11,85	4.8-28.5	32,0	14,83	17.2-46.8	13,8	10,96	2.8-24.8	20,0	12,72	7.3-32.7
Emergency r	Registered Nurses	118	4,1	56,0	8,96	47.1-65	51,9	9,01	42.9-60.9	34,5	8,58	26.0-43.1	64,0	8,66	55.3-72.6
	Physicians	24	0,8	87,0	13,47	73.5-100.4	65,2	19,05	46.2-84.3	34,8	19,05	15.7-53.8	65,2	19,05	46.2-84.3
	Other	17	0,6	46,7	23,72	23.0-70.4	22,2	19,76	2.5-42	36,4	22,87	13.5-59.2	52,9	23,73	29.2-76.7
General War	Registered Nurses	704	24,6	39,6	3,61	36.0-43.2	34,8	3,52	31.3-38.3	27,4	3,29	24.1-30.7	40,4	3,63	36.8-44.1
	Physicians	85	3,0	69,4	9,80	59.6-79.2	45,7	10,59	35.1-56.3	36,1	10,21	25.9-46.4	54,8	10,58	44.2-65.3
	Other	94	3,3	31,0	9,35	21.7-40.4	22,4	8,43	14.0-30.8	20,0	8,09	11.9-28.1	37,0	9,76	27.3-46.8
**ICU/CCU**	Registered Nurses	150	5,2	41,8	7,89	33.9-49.7	39,1	7,81	31.3-46.9	26,2	7,03	19.1-33.2	43,6	7,94	35.7-51.5
	Physicians	15	0,5	66,7	23,86	42.8-90.5	50,0	25,30	24.7-75.3	57,1	25,04	32.1-82.2	42,9	25,04	17.8-67.9
	Other	11	0,4	40,0	28,95	11.0-69.0	50,0	29,55	20.5-79.5	22,2	24,57	-2.3-46.8	54,5	29,43	25.1-84.0
OR/procedu	Registered Nurses	186	6,5	49,4	7,19	42.2-56.6	44,3	7,14	37.1-51.4	31,8	6,69	25.1-38.5	48,4	7,18	41.2-55.6
	Physicians	98	3,4	74,0	8,69	65.3-82.6	63,0	9,56	53.5-72.6	48,4	9,89	38.5-58.3	61,5	9,64	51.8-71.1
	Other	17	0,6	20,0	19,01	1.0-39.0	33,3	22,41	10.9-55.7	33,3	22,41	10.9-55.7	42,9	23,52	19.3-66.4
Outpatient u	Registered Nurses	435	15,2	33,3	4,43	28.8-37.7	32,7	4,41	28.3-37.2	20,8	3,81	16.9-24.6	34,7	4,47	30.2-39.1
	Physicians	138	4,8	55,1	8,30	46.8-63.4	40,2	8,18	32.0-48.3	41,6	8,22	33.4-49.8	39,0	8,14	30.8-47.1
	Other	182	6,4	32,1	6,78	25.3-38.8	15,1	5,20	9.9-20.2	23,0	6,12	16.9-29.1	25,3	6,32	19.0-31.7
Other	Registered Nurses	197	6,9	37,4	6,76	30.7-44.2	31,3	6,48	24.9-37.8	20,3	5,61	14.6-25.9	35,4	6,68	28.8-42.1
	Physicians	31	1,1	53,6	17,56	36.0-71.1	50,0	17,60	32.4-67.6	42,9	17,42	25.4-60.3	52,0	17,59	34.4-69.6
	Other	28	1,0	25,0	16,04	9.0-41.0	20,0	14,82	5.2-34.8	8,3	10,24	-1.9-18.6	26,7	16,38	10.3-43.0
**Total**	**Total**	**2864**	**100,0**	**42,6**	**1,81**	**40.8-44.4**	**36,6**	**1,76**	**34.8-38.3**	**27,7**	**1,64**	**26.1-29.4**	**40,6**	**1,80**	**38.8-42.4**
